# ﻿A new species of the genus *Luciogobius* Gill, 1859 (Teleostei, Oxudercidae) from Taiwan

**DOI:** 10.3897/zookeys.1206.118757

**Published:** 2024-07-09

**Authors:** Kuan-Hsun Chen, Te-Yu Liao

**Affiliations:** 1 Department of Oceanography, National Sun Yat-sen University, Kaohsiung, Taiwan National Sun Yat-sen University Kaohsiung Taiwan

**Keywords:** Actinopterygii, amphidromous, earthworm goby, interstitial habitat, taxonomy, western Pacific

## Abstract

A new species, *Luciogobiusopisthoproctus***sp. nov.**, is described based on 18 specimens collected from Daxi Creek (Yilan) and Babian Creek (Taitung) in Taiwan. The new species is characterized by having a yellowish body with scattered spots on the sides, a black blotch on the caudal fin, the absence of free pectoral-fin rays, and more than 40 vertebrae. The new species can be distinguished from congeners by the following combination of characters: AAA distance (anus to anal-fin origin) shorter than twice the body depth at anus, 4.2–7.2% of standard length (SL); pre-anus length 80.0–92.8% of pre-anal-fin length; snout length 39.7–62.7% of AAA distance; abdominal vertebrae 20–22; caudal vertebrae 20–22; first anal-fin pterygiophore usually inserted behind the second haemal spine.

## ﻿Introduction

The earthworm goby genus *Luciogobius* Gill, 1859 of the family Oxudercidae (sensu Nelson et al. 2016) comprises at least 16 valid species distributed along coasts of eastern Russia, Korea, China, Taiwan, Japan, and northern Vietnam (Lindberg and Krasyukova 1975; Chen et al. 2008; Cho and Choi 2014; Shibukawa et al. 2019, 2020; Ta et al. 2021; Koreeda and Motomura 2022). *Luciogobius* species mostly dwell in the intertidal zones or in estuaries that contain rocky substrates or sandy sediments (Yamada et al. 2009; Shibukawa et al. 2019; Koreeda and Motomura 2022). Their elongated bodies and finely segmented vertebral columns allow them to access various microhabitats (Yamada et al. 2009; Kondo and Kato 2018; Shibukawa et al. 2019, 2020). The most distinguishable characteristics of *Luciogobius*, which set them apart from other oxuderecid genera, are their specialized elongate and scaleless bodies with more vertebrae, degenerate eyes, and the absence of the first dorsal fin (Yamada et al. 2009; Nelson et al. 2016; Shibukawa et al. 2019, 2020). However, *Luciogobius* species lack notable morphological characters for distinguishing congeners (Shibukawa et al. 2019). After reviewing the taxonomy of *Luciogobius*, Shibukawa et al. (2019) grouped extant species into five species complexes based on morphological characters and named them after the earliest described species among each complex: *L.elongatus*, *L.grandis*, *L.guttatus*, *L.platycephalus*, and *L.pallidus* complexes (Shibukawa et al. 2019). However, it is still under debate whether each complex represents only one valid species, as there are still many undescribed and cryptic species (Shibukawa et al. 2019). Among these complexes, the *L.elongatus* complex is the largest and comprises *L.adapel* Okiyama, 2001, *L.elongatus* Regan, 1905, *L.parvulus* (Snyder, 1909), *L.punctilineatus* Koreeda & Motomura, 2022, and nine unnamed species, i.e. *Luciogobius* spp. 8–16, and this complex can be distinguished from *L.grandis*, *L.guttatus*, *L.pallidus* complexes by having an AAA distance of more than half of the body depth at the anus (vs less than half of body depth). It can be further distinguished from *L.guttatus* and *L.pallidus* complexes by having the first anal-fin pterygiophore insertion behind the first haemal spine (vs before the first haemal spine). It can also be separated from *L.grandis* and *L.platycephalus* complexes by having the anteriormost pleural rib attached to the third position (vs second) of the abdominal vertebra (Shibukawa et al. 2019). In the present study, several specimens of an undescribed species of the *L.elongatus* complex were collected from eastern Taiwan. The new species is described here based on morphological and molecular characters.

## ﻿Material and methods

**Figure 1.**
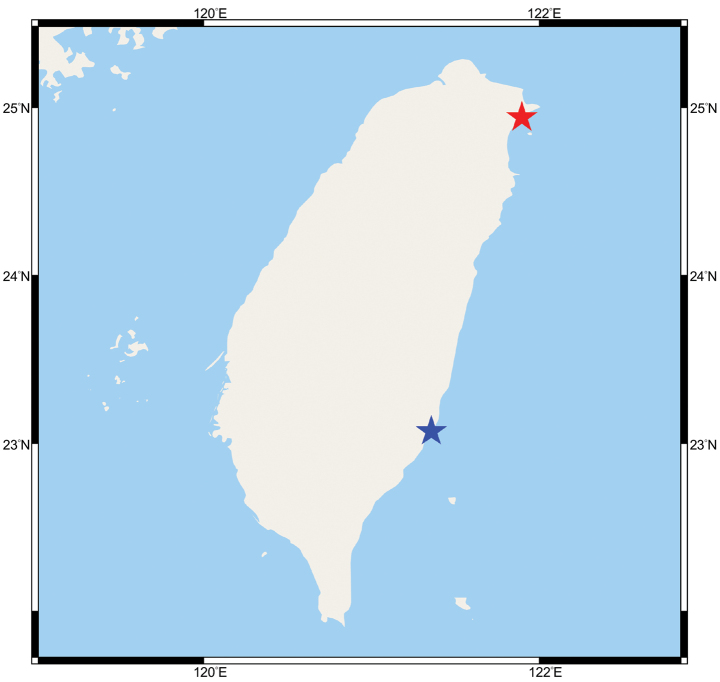
Map of Taiwan showing the collection localities of *Luciogobiusopisthoproctus* sp. nov. Red star, Daxi Creek; blue star, Babian Creek.

**Figure 2.**
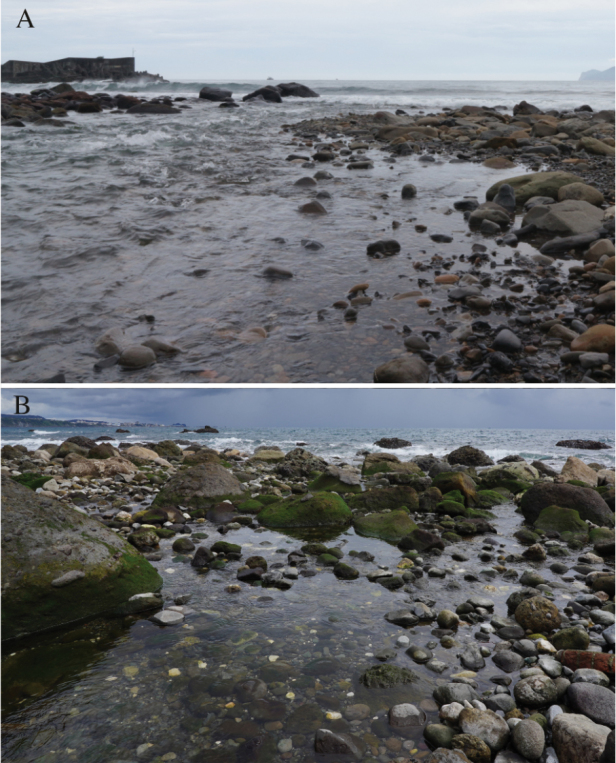
Habitats of *Luciogobiusopisthoproctus* sp. nov. **A** river mouth of the Daxi Creek, Yilan County, Taiwan **B** river mouth of the Babian Creek, Taitung County, Taiwan.

Specimens were collected from the mouths of the Daxi Creek, Yilan County, and Babian Creek, Taitung County (Figs 1, 2) using a hand-net in water 0–30 cm deep during low tide. The substrate was gravel (5–8 mm in diameter). After sampling, each specimen was photographed, muscle tissues or fin clips were collected, preserved in 95% ethanol solution, and stored at −20 °C for molecular analysis. The specimens were then fixed in 10% neutral buffered formalin and transferred to 70% ethanol solution for permanent preservation. The specimens are deposited in the Department of Oceanography, National Sun Yat-sen University, Kaohsiung (**DOS**), the Academia Sinica Institute of Zoology, Taiwan (**ASIZP**), and the National Museum of Marine Biology and Aquarium (**NMMB-P**), Pingtung. We examined one *L.elongatus* specimen from Osaka Museum of Natural History (**OMNH-P**) for comparison. Meristic counts and morphometric measurements follow Shibukawa et al. (2019), except for the following characters which follow Koreeda and Motomura (2022): AAA was measured from the posterior margin of the anus to the anal-fin origin; body depths were taken at the pelvic-fin origin, anus and anal-fin origin; counts of the abdominal and caudal vertebrae. Other abbreviations: SL, standard length; HL, head length. The series of cephalic sensory papillae on the cheek was based on Shibukawa et al. (2020). The alcian blue–alizarin red staining method (Dingerkus and Uhler 1977) and radiographs were used for counts of the dorsal and anal fin rays, pterygiophores, and vertebrae for all the type specimens. Cyanine blue was used for papillae illustration (Saruwatari and Andrés López 1997).

The GeneMark Easy Tissue & Cell Genomic DNA Purification kit was used for DNA extraction from muscle tissues or fin clips following the manufacturer’s protocol. The partial mitochondrial 12S ribosomal RNA (12S) (133 bp) was amplified for molecular analysis. Polymerase chain reactions (PCR) were performed using a 25 μl volume containing 16.47 μl double distilled water, 3.0 μl of 10X Taq Buffer, 2.0 μl of dNTP mixture at 10 mM, 1.2 μl of each forward and reverse primers at 5 μM, 0.125 μl of Pro Taq Plus DNA polymerase, and 1.0 μl of template DNA. In some cases, PCR were also performed in a 25 μl volume consisting of 9.5 μl double distilled water, 12.5 μl SuperRed PCR Master Mix (2×), 1.0 μl each of forward and reverse primers at 5 μM, and 1.0 μl of template DNA. The mitochondrial 12S rRNA gene was amplified using universal primers MiFishU-F and MifishU-R (Miya et al. 2015), with PCR thermal conditions consisting of an initial denaturation of 95 °C for 4 min, followed by 30 cycles of 94 °C for 40 s of denaturation, 60 °C for 20 s of annealing and 72 °C for 20 s of initial extension, and final extension at 72 °C for 4–10 min. The PCR products were visualized in 2% agarose gels, purified using SAP-Exo Kit (Jena Bioscience, Jena, Germany) and sent to Genomics S&T (Taipei, Taiwan) for sequencing. Sequences were assembled and edited using SeqMan Pro v. 11.1.0 (DNASTAR Inc., Madison, WI, USA). The obtained 12S sequences were submitted to GenBank under accession numbers OR871675–OR871682 and OR879784. Sequences of *L.elongatus* (LC499453, LC500721, LC579249, LC579251, MH682217), *L.parvulus* (LC717554, LC579254), *L.punctilineatus* (LC677209–LC677214), *L.* sp. 8 (LC774564, LC772867), *L.* sp. 9 (LC499443, LC579355), *L.* sp. 10 (LC499449, LC579264), *L.* sp. 11 (LC579262, LC579278), *L.* sp. 12 (LC579265), *L.* sp. 13 (LC499441, LC579281), *L.* sp. 14 (LC499442, LC499450), *L.* sp. 15 (LC579275, LC579349), *L.* sp. 16 (LC722561, LC722563) were downloaded from GenBank (12S sequence of *L.adapel* is not available). A single sequence of *Inukoma* (LC579243) was used as an outgroup for tree rooting. Sequences were aligned using MUSCLE (Edgar 2004) alignment in SeqMan v. 11.1.0, and substitution saturation of all sequences was tested using DAMBE v. 6.3.17 (Xia and Xie 2001). The Tamura-3 + G model was selected to reconstruct a maximum-likelihood (ML) tree (Felsenstein 1981) using MEGA v. X (Kumar et al. 2018) with a 1,000 replicates bootstrapping estimation for branch supports. The pairwise distance (*p*-distance) between each pair of the species was calculated using the Kimura 2-parameter (K2P) model in MEGA v. X (Kumar et al. 2018).

## ﻿Results

### ﻿ Luciogobius opisthoproctus sp. nov.

Taxon classificationAnimaliaPerciformesOxudercidae

EEF6E028-0E05-5326-8C07-92A5E5D130FC

https://zoobank.org/D65F19DD-CC3D-486F-BBC7-7DE41D55226E

**Figure 3.**
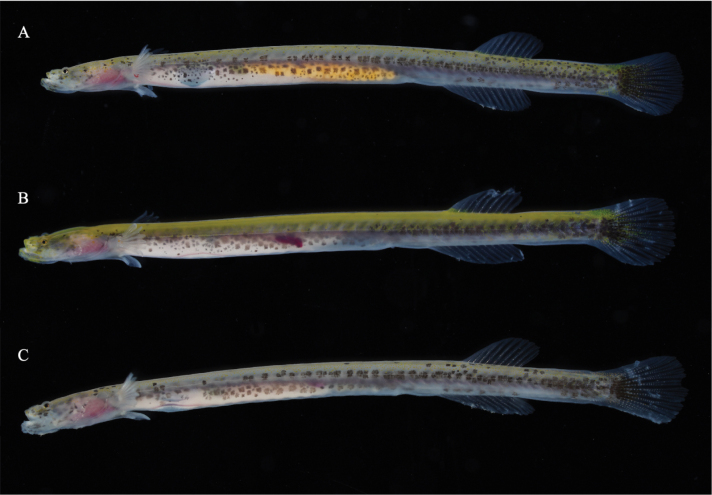
Fresh specimens of *Luciogobiusopisthoproctus* sp. nov. **A** holotype, ASIZP0081790, 25.4 mm SL, Daxi Creek, Yilan County, Taiwan **B** paratype, ASIZP0081793, 23.8 mm SL, Daxi Creek, Yilan County **C** paratype, NMMB-P39322, 21.4 mm SL, Daxi Creek, Yilan County, Taiwan.

**Figure 4.**
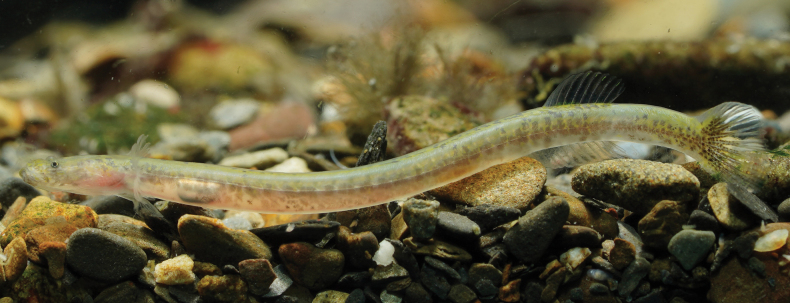
Live *Luciogobiusopisthoproctus* sp. nov. NMMB-P39326, paratype, 27.5 mm SL, Daxi Creek, Yilan County, Taiwan.

**Figure 5.**
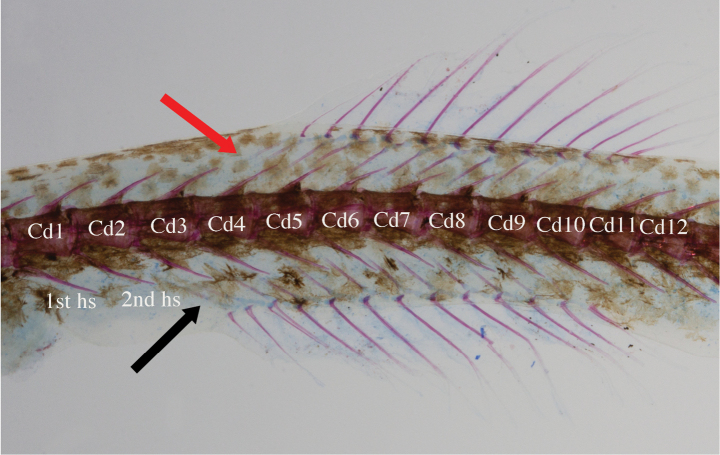
The alcian blue–alizarin red stained specimens of *Luciogobiusopisthoproctus* sp. nov. DOS09993-2, paratype, 24.9 mm SL, Daxi Creek, Yilan County, Taiwan, coll. K.H. Chen, 16 February 2023. Cd indicates caudal vertebrae; Hs indicates haemal spine of caudal vertebrae. Both abbreviations are numbered. The red arrow indicates the first dorsal pterygiophore insertion; the black arrow indicates the first anal-fin pterygiophore insertion.

Figs 3 , 4 , 5 New English name: Taiwan Earthworm Goby

#### Holotype.

ASIZP0081790, 25.8 mm SL, Taiwan, Yilan County, mouth of Daxi Creek, 24°56.48'N, 121°53.72'E, coll. K.H. Chen, 16 February 2023.

#### Paratypes.

17 specimens (20.6–28.1 mm SL). ASIZP0081791, 22.9 mm SL, ASIZP0081792, 22.9 mm SL, ASIZP0081793, 23.8 mm SL, DOS09993-1, 25.1 mm SL, DOS09993-2, 24.9 mm SL, DOS09993-3, 26.8 mm SL, DOS09993-4, 22.8 mm SL, DOS09993-5, 20.6 mm SL, DOS09993-6, 22.8 mm SL, NMMB-P39322, 22.7 mm SL, NMMB-P39323, 21.44 mm SL, NMMB-P39324, 21.8 mm SL, NMMB-P39325, 23.1 mm SL, NMMB-P39326, 27.5 mm SL, collected with holotype. ASIZP0081794, 27.3 mm SL, DOS09994-1, 24.3 mm SL, NMMB-P39327, 28.1 mm SL, Taiwan, Taitung County, river mouth of Babian Creek, 23°04.44'N, 121°21.38'E, coll. K.H. Chen, 21 March 2023.

#### Diagnosis.

*Luciogobiusopisthoproctus* sp. nov. is diagnosed by the following combination of characters: total vertebrae 41–43; free pectoral-fin rays absent; second dorsal-fin rays 9–12 (usually 11); anal-fin rays 11–14 (11 or 12); pectoral-fin rays 8–12 (10 or 11); pelvic-fin length more than 50% of pectoral-fin length; AAA distance 4.2–7.2% (mean 5.7%) of SL, 72.1–129.7% (mean 99.9%) of body depth at anus; snout length 39.7–62.7% (mean 52.8%) of AAA distance; pre-anus distance 80.0–92.8% (mean 88.6%) of pre-anal-fin length; and anterior-most pterygiophore of anal fin inserted behind the second haemal spine (Fig. 5).

#### Description.

Morphometric measurements and meristic counts are given in Table 1. Body elongate, anteriorly cylindrical, and posteriorly compressed. Head narrowly depressed. Anterior nostril a pair of short tubes; posterior nostril round. Lower jaw slightly projecting; mouth oblique; maxilla extends posteriorly to vertical at front margin of eye. Gill opening narrow, extending from middle of pectoral-fin base to level with posterior end of jaw. Interorbital space narrower than snout length, side compressed, anteriorly swollen. A longitudinal dermal ridge present from below anterior nostril extending to behind eye. Occipital region slightly turgid (swollen) dorsally and laterally. First dorsal fin absent. Origin of second dorsal fin slightly posterior to origin of anal fin. First and second rays of second dorsal fin spinous, remaining rays soft and segmented; posterior margin of second dorsal fin rounded; second dorsal-fin rays 9–12 [9 (3), 10 (5), 11 (8)*, 12 (2)] (* for the number including holotype). Anal fin slightly posterior to anus; first and second rays of anal fin spinous, remaining rays soft and segmented; posterior margin of anal fin rounded; anal fin rays 11–14 [11 (5), 12 (7)*, 13 (4), 14 (2)]. Pectoral-fin fan-shaped and free pectoral-fin rays absent, pectoral-fin relatively small, about 5.5–8.8% of SL; pectoral-fin rays 8–12 [8 (1), 9 (1), 10 (5)*, 11 (6), 12 (5)]. Caudal fin rounded. Pelvic fins round with frenum and complete membrane; pelvic-fin rays I, 5. Segmented caudal-fin rays 15–17 [15 (5), 16 (9), 17 (4)*]. First dorsal pterygiophore inserted between 25^th^ and 26^th^ vertebrae; last dorsal pterygiophore inserted between 31^st^ and 32^nd^ vertebrae; first anal fin pterygiophore inserted behind second haemal spine; insertion pattern not changing with numbers of fin rays; abdominal vertebrae 20–22 (usually 22), caudal vertebrae 20–22 (usually 21).

**Table 1.** Morphometric measurements and meristic counts of *Luciogobiusopisthoproctus* sp. nov.

	**Holotype**	**Paratypes**	**Paratypes**
		**Daxi Creek (14)**	**Babian Creek (3)**
Standard length (SL; mm)	25.4	20.6–27.5	24.3–28.1
Count			
D_2_ elements	11	9–12	9–11
A elements	12	11–14	11–13
P_2_ elements	6	6	6
P_1_ elements	10	8–12	10–12
C segmented elements	15	16–17	15–16
V	21+22	20–22+20-22	22+21
First D pterygiophore insertion	25–26	25–26	25–26
First A pterygiophore insertion behind	2^nd^ hs	2^nd^ hs	2^nd^ hs
Measurement (%SL)			
HL	14.1	12.7–18.7	13.4–16.6
HD	5.8	3.6–6.5	5.4–6.0
HW	7.1	5.4–8.8	6.8–7.4
SNL	3.1	2.5–3.4	2.6–3.0
IOW	2.9	1.5–2.9	1.7–2.6
BD at P_2_ origin	6	4.3–5.9	5.4–5.8
BD at AN	6.3	4.8–6.5	5.2–6.9
BD at A origin	6.4	4.9–6.3	5.0–6.5
PANL	66.4	60.5–66.4	63.2–65.0
AAA distance	6.6	4.2–7.2	5.0–6.3
CPD	6.6	3.9–5.9	4.1–5.0
CPL	15.8	12.6–16.8	13.2–16.4
PAL	73.8	68.9–73.5	70.0–72.6
PP_2_L	15.7	15.6–19.0	14.9–16.0
D_2_L	11	8.3–12.2	9.2–12.3
AL	14.7	10.8–16.5	9.2–13.5
P_1_L	7.4	5.5–8.8	6.4–7.8
P_2_L	4.3	3.8–6.1	4.2–5.2
CL	10.4	7.8–15.4	11.1–12.4
PD_2_L	73.9	69.9–77.8	73.3–74.8
Measurement (% HL)			
HD	40.8	26.0–46.5	33.8–40.5
HW	50.6	39.5–65.3	40.7–52.9
SNL	22.1	16.2–24.8	15.6–20.4
ED	6.4	4.5–10.1	3.6–7.1
IOW	20.4	10.7–19.6	12.5–15.4
SNL (% AAA distance)	52.7	39.7–62.7	45.3–51.5
PANL (% PAL)	90	80.0–92.4	88.2–92.8
P_2_L (% P_1_L)	58.5	57.5–84.9	62.1–69.7
AAA distance (% BD at anus)	93.2	72.1–129.7	72.3–121.4

D_2_: second dorsal-fin; A: anal-fin; P_2_: pelvic-fin; P_1_: pectoral-fin; C: caudal-fin; V: vertebrae; D: dorsal; hs: haemal spine; P_1_L: pectoral-fin length; BD: body depth; AN: anus; PAL: pre-anal-fin length; AAA distance: distance between anus and anal-fin origin; CPD: caudal-peduncle depth; CPL: caudal-peduncle length; PP_2_L: pre-pelvic-fin length; D_2_L: second dorsal-fin length; AL: anal-fin length; P_1_L: pectoral-fin length; P_2_L: pelvic-fin length; CL: caudal-fin length; PD_2_L: pre-second dorsal-fin length; PANL: pre-anus length; ED: eye diameter.

#### Cephalic sensory system.

**Figure 6.**
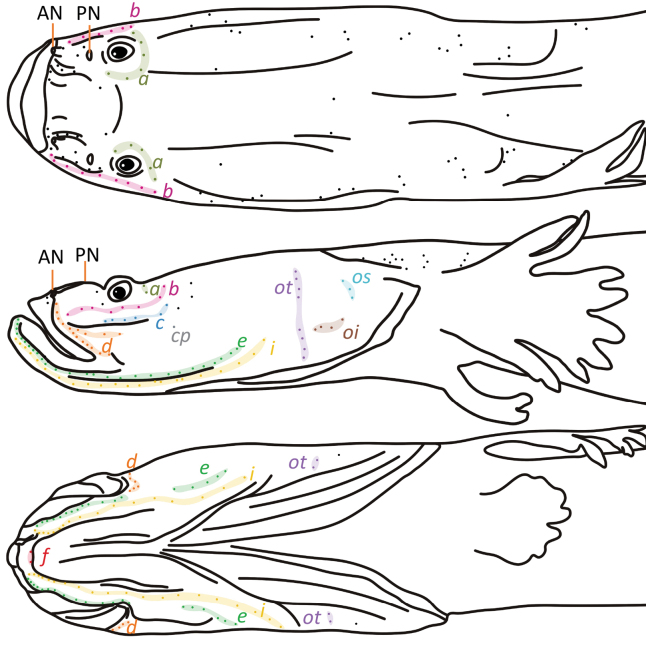
Head cephalic sensory system of *Luciogobiusopisthoproctus* sp. nov. ASIZP0081790, holotype, 25.4 mm SL, Daxi Creek, Yilan County, Taiwan. AN, Anterior nostril; PN, Posterior nostril. Color-marked spots with letters indicate the papillae and the name of each row.

The series of cephalic sensory papillae on cheek are illustrated in Fig. 6. Row *a* situated behind eye, extending shortly upwards to orbital area. Row *b* extending from margin behind eye to upper lip. Row *c* starting from posterior of dermal ridge to anterior margin of eye. Single spot *cp* situated under row *c*. Row *d* starting from posterior margin of upper lip to anterior nostril. Row *e* extending from lower margin of preopercle to upper margin of lower jaw. Row *i* extending along weak flap on lower margin of lower jaw to lower part of preopercle. Row *f* in posterior of symphysial flap on chin. Row *oi* longitudinal, row *ot* and *os* running vertically. Row *oi*, *os*, *ot* separated from each other.

#### Coloration.

Body background white, dorsally yellowish, and translucent ventrally. A discontinuous row of black spots on side of body from pectoral-fin to caudal-fin base. Scattered black spots internally embedded in the abdomen and visible through the semi-transparent muscle. Scattered black spots on dorsal surface, highly dense in some individuals. Rays and membranes of dorsal-, pectoral-, pelvic-, and anal-fins transparent and yellowish at base. Caudal fin transparent, with a black, rounded blotch at base. Specimens preserved in 70% ethanol whitish, with the same pattern of black spots when alive.

#### Distribution and habitat.

The new species is currently only known from northeastern and southeastern Taiwan. This species mainly inhabits shallow gravel creeks near coastal river mouths.

#### Etymology.

The specific name *opisthoproctus* is from the Greek words *opisthe* (behind) and *proktos* (anus), in allusion to the posteriorly positioned anus (shorter AAA distance).

#### Molecular analysis.

**Figure 7.**
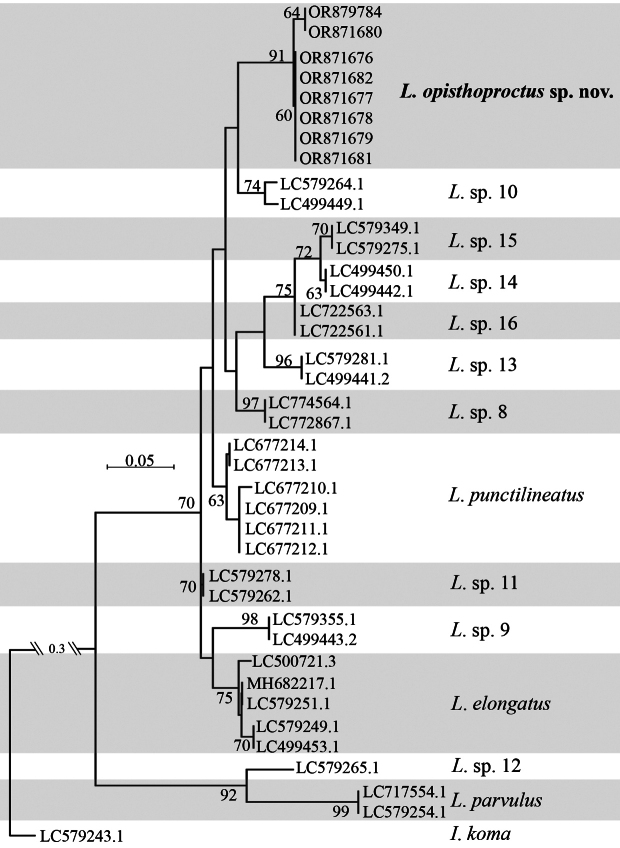
Maximum-likelihood (ML) tree based on 12S ribosomal RNA gene sequences (133 bp) using Tamura-3+G model with 1,000 bootstrap replications. Numbers of branches indicate bootstrap values higher than 60%.

Thirty-nine sequences (133 bp) from 14 species were obtained and used to reconstruct an ML tree (Fig. 7), with *Inukoma* as the outgroup. In the topology of the ML tree, a monophyletic clade consisting of *L.opisthoproctus* sp. nov. was revealed. *Luciogobiusopisthoproctus* sp. nov. and *L.* sp. 10 are sister species, and these two species are a sister group of a clade comprising *Luciogobius* spp. 8, 13, 14, 15, and 16. The genetic distances between the new species and other members of *L.elongatus* complex are shown in Table 2.

**Table 2.** Pairwise sequence differences (%: *p*-distance) of 133 bp of 12S ribosomal RNA gene among species of *Luciogobiuselongatus* complex. The hyphen “–” indicates that only one sequence is available.

		1	2	3	4	5	6	7	8	9	10	11	12	13	14
1	*L.opisthoproctus* sp. nov. (8)	0–0.8													
2	*L.elongatus* (5)	5.9–7.6	0–1.8												
3	*L.parvulus* (2)	15.1–16.0	12.6–14.3	0											
4	*L.punctilineatus* (6)	5.9–7.6	4.2–6.7	1.2–1.3	0–1.7										
5	*L.* sp. 8 (2)	5.9–6.7	4.2–5.9	1.3	4.2–5.9	0									
6	*L.* sp. 9 (2)	4.2–17.6	4.2–5.0	13.4–14.3	4.2–5.0	7.6	0								
7	*L.* sp. 10 (2)	4.2–17.6	5.0–7.6	12.6	5.0–7.6	5.0	7.6	1.7							
8	*L.* sp. 11 (2)	4.2–5.9	2.5–3.4	12.6	2.5–3.4	4.2	3.4	1.7–4.2	0						
9	*L.* sp. 12 (1)	12.6–13.4	11.8–13.4	6.7	11.8–13.4	11.8	13.4	10.9–12.6	10.1	–					
10	*L.* sp. 13 (2)	4.2–17.6	3.4–4.2	14.3	3.4–4.2	3.4	6.7	5.0–5.9	3.4	12.6	0				
11	*L.* sp. 14 (2)	5.0–8.4	5.9–6.7	14.3	5.9–6.7	5.0	9.2	5.6–6.7	5.9	13.4	4.2	0			
12	*L.* sp. 15 (2)	8.4–9.2	5.0–5.9	14.3	5.0–5.9	5.9	8.4	6.7–7.6	5.0	13.4	5.0	8	0		
13	*L.* sp. 16 (2)	6.7–7.6	5.0–5.9	14.3	5.0–5.9	4.2	8.4	5.0–5.9	5.0	13.4	3.4	1.7	2.5	0	
14	*I.koma* (1)	16.8–17.6	16.8–18.5	17.6	16.0–16.8	16.0	18.5	17.6–18.5	16.8	16.8	16.0	1.6	15.1	16.0	–

Numbers in paracenteses for numbers of sequences.

#### Remarks.

Based on the morphological approach, *L.opisthoproctus* sp. nov. belongs to the *L.elongatus* complex (Shibukawa et al. 2019) because of the following characters: absence of free pectoral-fin rays, AAA 72.1–129.7% of body depth at anus, vertebrae 41–43, anterior pleural attaching to third abdominal vertebra, first anal-fin pterygiophore inserted behind second haemal spine, and first to second rays of dorsal- and anal-fin unbranched and spine-like. The new species is most similar to *L.punctilineatus* and *L.* sp. 11 in having yellowish coloration, a black blotch on the caudal-fin, a longitudinal line composed of scattered black spots on the sides of body, and similar_numbers of dorsal-, anal-, pectoral-fin elements. However, *L.opisthoproctus* sp. nov. can be distinguished from *L.punctilineatus* and *L.* sp. 11 by the shorter AAA distance (4.2–7.2% SL vs 11.4–16.9% in *L.punctilineatus*; 9.1–10.4% in *L.* sp.11); longer pre-anus length (80.0–92.8% of pre-anal-fin length vs 75.0–82.0% in *L.punctilineatus*; 84.4–88.8% in *L.* sp.11); longer snout length (39.7–62.7% of AAA distance vs <34.7% in *L.punctilineatus*; 36.7–44.8% in *L.* sp.11); more abdominal vertebrae (20–22 vs 16–18 in *L.punctilineatus*; 17–18 in *L.* sp.11); fewer caudal vertebrae (20–22 vs 22–24 in *L.punctilineatus*; 22–25 in *L.* sp.11); first anal fin pterygiophore usually inserted behind the second haemal spine (vs fifth in *L.punctilineatus*; fourth in *L.* sp.11) (Table 3). The new species can be further distinguished from *L.punctilineatus* in having the AAA distance less than twice the body depth at the anus (vs about twice of the body depth at anus).

**Table 3.** Morphometric measurements and meristic counts between *Luciogobiusopisthoproctus* sp. nov. and three morphologically similar species.

	*L.opisthoproctus* sp. nov.^1^	*L.punctilineatus* ^2^	*L.* sp. 10^3^	*L.* sp. 11^2^
Ab V	20–22	16–18	14	17–18
Cd V	20–22	22–24	22	22–25
First A pterygiophore insertion behind	2^nd^ hs	4–6 (5)^th^ hs	N/A	4^th^ hs
Measurement				
HL (% SL)	12.7–18.7% (14.5%)	12.7–16.6% (N/A)	19.5–19.7% (N/A)	N/A
AAA distance (% SL)	4.2–7.2% (5.7%)	11.4–16.9% (13.3%)	N/A	9.1–10.4% (9.6%)
AAA distance (% BD at AN)	72.1–129.7% (99.9%)	Twice BD	N/A	N/A
SNL (% AAA)	39.7–62.7% (52.8%)	19.2–34.7% (26.4%)	N/A	36.7–44.8% (40.9%)
PANL (% PAL)	80.0–92.8% (88.6%)	75.0–82.0% (79.3%)	N/A	84.4–88.8% (86.1%)
P_2_L (% P_1_L)	57.5–84.9 (69.4%)	42.2%*	N/A	N/A
Reference	Present study	Koreeda et al. 2022	Shibukawa et al. 2019	Koreeda et al. 2022

* Indicates only one specimen is available. N/A: not available. Abbreviation: Ab V: abdominal vertebrae; Cd V: caudal vertebrae; A: anal fin; hs: haemal spine; HL: head length; SL: standard length; AAA distance: distance between anus and anal-fin origin; BD: body depth; AN: anus; SNL: snout length; PANL: pre-anus length; PAL: pre-anal-fin length; P_1_L: pectoral-fin length; P_2_L: pelvic-fin length. 1 for present study; 2 for Koreeda et al. 2022; 3 for Shibukawa et al. 2019.

Based on the molecular analysis, the topology showed that there is a sister-species relationship between the new species and *L.* sp. 10. However, *L.opisthoproctus* sp. nov. can be distinguished from *L.* sp. 10 (Table 3) in having a smaller HL (12.7–18.7% vs 19.5–19.7% SL), more abdominal vertebrae (20–22 vs 14) and more total vertebrae (41–43 vs 36) (Table 4). *Luciogobiusopisthoproctus* sp. nov. can be easily distinguished from *L.adapel* and *L.parvulus* by the presence of a pelvic fin (vs absence) and further distinguished from *L.adapel* by the presence of a second dorsal and anal fin (vs absence); from *L.elongatus*, *L.* sp. 8, and *L.* sp. 9 by more dorsal-fin rays (9–12 vs usually less than 9) and anal-fin rays (11–14 vs usually less than 11), and further distinguished from *L.elongatus* and *L.* sp. 9 by presence of a well-developed frenum on the pelvic fin (vs absence). The new species can also be distinguished from *Luciogobius* spp. 12–16 by absence of free pectoral-fin rays (vs presence). It can be further distinguished from *L.* sp. 12 and *L.* sp. 13 by having more vertebrae in total (41–43 vs 38–39), and from *Luciogobius* sp. 14–16 by presence of a black blotch on the caudal-fin (vs absence) (Table 4). *Luciogobius* sp. 3 (sensu Maeda et al. 2008) is also similar to *L.opisthoproctus* sp. nov. in coloration, morphometric measurements, and meristic counts. However, due to the limited information from only one juvenile provided by Maeda et al., further study is needed to verify the relationship of these sepcies. Species within the *L.elongatus* species complex, as defined by Shibukawa et al. (2019), exhibit a unique combination of characters that differentiate them from species in other complexes. However, the presence of a single free pectoral-fin ray of *L.parvulus* and *L.* spp. 12–16 of the *L.elongatus* complex is not constant. This trait is apparently not a reliable character to distinguish *L.elongatus* complex from *L.guttatus* complex (vs presence of a free pectoral-fin ray).

**Table 4.** Selected characters of the species of *Luciogobiuselongatus* complex.

	D2 element	A element	P_1_ element	Ab V	Cd V	V	Free P_1_ rays	P_2_	P_2_ frenum	Sources
*L.adapel*	Absent	Absent	9–10	23	26–27	49–50	Absent	Absent	ND	1
*L.opisthoproctus* sp. nov.	9–12	11–14	8–12	20–22	20–22	41–43	Absent	Present	W-D	2
*L.elongatus*	6–9	8–10	7–10	19–21	21–23	42–44	Absent	Present	ND	3
*L.parvulus*	10–12	11–13	11–13	19–21	22–24	41–44	Present*	Absent	ND	4
*L.punctilineatus*	10–12	12–14	8–12	16–18	22–24	39–42	Absent	Present	W-D	4
*L.* sp. 3	8–10	11–12	9–10	21–23	19–21	42	Absent	Present	W-D	5
*L.* sp. 8	7–8	8–10	9–11	20–21	20–23	41–43	Absent	Present	W-D	3
*L.* sp. 9	7–9	8–11	8–10	20–22	21–23	42–45	Absent	Present	ND	3
*L.* sp. 10	8	11	11–12	14	22	36	Absent	Present	W-D	3
*L.* sp. 11	12–13	14–15	8–9	17–18	22–25	40–43	Absent	Present	W-D	3
*L.* sp. 12	10–12	10–11	14	18–19	20–21	38–39	Present	Present	W-D	3
*L.* sp. 13	9–11	12–13	13–15	16–17	21–23	38–39	Present	Present	W-D	3
*L.* sp. 14	10–13	12–15	12–14	19–21	21–23	41–43	Present	Present	W-D	3
*L.* sp. 15	11–13	13–14	11–14	18–20	21–23	39–42	Present	Present	W-D	3
*L.* sp. 16	11–13	12–14	10–13	20–21	22–23	22–23	Present*	Present	W-D	3

Abbreviation: D_2_: second dorsal-fin; A: anal-fin; P_1_: pectoral-fin; P_2_: pelvic-fin; Ab V: abdominal vertebrae; Cd V: caudal vertebrae; V: vertebrae; ND: not developed; W-D: well-developed. *=sometimes absent. 1 for Okiyama, 2001; 2 for present study; 3 for Shibukawa et al. 2019; 4 for Koreeda and Motomura 2021; 5 for Maeda et al. 2008.

**Figure 8.**
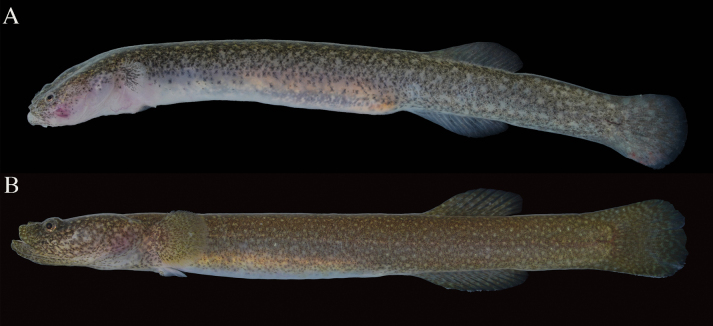
Photos of fresh specimens coexisting with *Luciogobiusopisthoproctus* sp. nov. **A***L.grandis*, DOS09990-2, 53.7 mm SL, Hualien River, Hualien County, Taiwan **B***L.guttatus*, DOS09988-1, 54.7 mm SL, Jinghuken, Yilan County, Taiwan.

*Luciogobiusopisthoproctus* sp. nov. sometimes co-occurs with *L.grandis* Arai, 1970 and *L.guttatus* Gill, 1859 near the mouths of streams. However, *L.opisthoproctus* sp. nov. can be morphologically distinguished by its yellowish body color, differing from the bronze color in *L.grandis* and the brown color in *L.guttatus* (Fig. 8). Additionally, it can also be readily distinguished from other two species by several distinctive features, such as a transparent caudal fin with one black blotch on its base (vs opaque), a pair of fan-shaped pectoral-fin (vs triangular in *L.grandis* and round in *L.guttatus*), absence of pigmentation on pectoral-, anal-, and second dorsal fins (vs presence), and unbranched first to second rays of dorsal- and anal-fins (vs only first ray unbranched).

#### Comparative material.

*L.elongatus*: one specimen, OMNH-P14170, 37.1 mm SL, Nagasaki Coast, Fuke, Misaki, Sennan, Osaka, Japan, coll. Kanai, M., 27 May 2001. *L.grandis*: ten specimens, DOS09990, 30.5–54.1 mm SL, Hualien River, Hualien County, Taiwan, coll. K. H. Chen, 31 January 2022. *L.guttatus*: ten specimens, DOS09988, 38.4–58.7 mm SL, Jinhuken, Yilan County, Taiwan, coll. K.H. Chen, 10 August 2021.

## Supplementary Material

XML Treatment for Luciogobius opisthoproctus

## ﻿Additional information

### Data availability

All of the data that support the findings of this study are available in the main text.
